# Glycemic Profiles and Hypoglycemia Awareness Among Pregnant Women with Gestational and Pre-existing Diabetes Referred to a Tertiary Center in Sulaimaniyah-Iraq in 2024

**DOI:** 10.5812/ijem-153529

**Published:** 2024-12-21

**Authors:** Jamal Mahmood Salih

**Affiliations:** 1 Department of Medical Education, College of Medicine, University of Sulaimani, Sulaimaniyah, Kurdistan Region, Iraq; 2 Maternity Diabetes Center, Maternity Teaching Hospital, Sulaimaniyah, Kurdistan Region, Iraq

**Keywords:** Glycemic Profile, Hypoglycemia Awareness, Gestational Diabetes, Hyperglycemia in Pregnancy

## Abstract

**Background:**

Hyperglycemia in pregnancy (HIP) comprises gestational diabetes mellitus (GDM) and pre-existing diabetes; type 1 diabetes (T1DM), type 2 diabetes (T2DM), and undetermined diabetes. Hyperglycemia in pregnancy leads to fetal and maternal complications.

**Objectives:**

To observe and compare glycemic profiles (GP) and hypoglycemia awareness (HA) in women with GDM and pre-existing diabetes.

**Methods:**

This prospective observational comparative study enrolled women with HIP registered at Sulaimani Maternity Teaching Hospital from January to April 2024. Self-monitoring blood glucose (SMBG) was used to document GP through mean blood glucose (MBG) analysis and the proportions of hyperglycemic, euglycemic, and hypoglycemic records. The Gold score was used to assess HA. Statistical analysis was conducted using SPSS version 27.0, employing chi-square, Mann-Whitney, Fisher's exact test, Kruskal-Wallis test, ANOVA, and independent *t*-tests. A P-value of ≤ 0.05 was considered significant.

**Results:**

One hundred patients were included in the final analysis. Half of the women were over 35 years old, 53% had GDM, and 47% had pre-existing diabetes. The MBG levels at fasting, 1-hour post-breakfast, and post-dinner were significantly highest in T1DM and lowest in GDM, while the levels were similar after lunch. Compared with pre-existing diabetes, women with GDM had a significantly greater proportion of euglycemic records and a lesser proportion of hyperglycemic and hypoglycemic records. Daily insulin requirements were significantly higher in women with pre-existing diabetes than in those with GDM (0.52 ± 0.35 vs 0.24 ± 0.12 units/kg, respectively, P < 0.001). Hypoglycemia episodes (HE) were 5.7 vs 1.83 events/patient/month in pre-existing diabetes vs GDM, respectively (P = 0.002). Using the Gold score to determine HA, 40% of T1DM patients had reduced HA, 40% had borderline HA, while 20% of T1DM and patients with other types of diabetes had normal HA (P < 0.001).

**Conclusions:**

Women with GDM had a significantly more stable GP, fewer HE, and lower insulin requirements than those with pre-existing diabetes. Type 1 diabetes patients had the most unstable GP, with significantly higher proportions of hyperglycemic and hypoglycemic records and reduced HA.

## 1. Background

Hyperglycemia in pregnancy (HIP) is a common metabolic disease worldwide. Hyperglycemia in pregnancy has been classified into gestational diabetes mellitus (GDM) and pre-existing diabetes. GDM is hyperglycemia diagnosed during the second half of pregnancy, while pre-existing diabetes refers to overt diabetes diagnosed before pregnancy, including type 1 diabetes (T1DM), type 2 diabetes mellitus (T2DM), and other types, such as monogenic diabetes (1). In 2021, the prevalence of GDM was estimated to be 14% globally and 27.6% in the Middle East and North Africa, while pre-existing diabetes affects < 2.4% of pregnancies (2). The global prevalence of GDM and pre-existing diabetes has increased in recent decades, especially among certain ethnic groups and in Middle Eastern countries (3).

Hyperglycemia in pregnancy can lead to maternal, obstetrical, and fetal complications. Diabetes increases the risk of abortion, preterm labor, fetal macrosomia, large for gestational age, congenital anomalies, and neonatal hypoglycemia (4, 5). However, these complications appear to be more common among women with pre-existing diabetes than those with GDM, particularly when hyperglycemia is not appropriately treated (5). Nevertheless, these complications can be minimized by pre-pregnancy counseling aimed at maintaining near-normal glycated hemoglobin (HbA1c), screening for complications, discontinuing unsafe medications and behaviors, and consuming folic acid in women with pre-existing diabetes, as well as maintaining tight glycemic control throughout the pregnancy (4-6).

Management of hyperglycemia includes dietary and lifestyle interventions, as well as antidiabetic medications. A proper diet and physical activity can be sufficient for a proportion of women with GDM. In contrast, the remaining proportion of women with GDM and those with pre-existing diabetes require medications to maintain euglycemia (7). Insulin is the preferred treatment for diabetes during pregnancy; however, there is no substantial evidence to recommend the best insulin regimen (8). Metformin can be prescribed if the patient refuses insulin or if insulin is intolerable. Metformin is not teratogenic; however, it can cross the placenta, increasing the risk of low fetal weight and pre-eclampsia (8, 9).

Tight glycemic control is crucial in the prevention of diabetes complications; however, maintaining euglycemia is neither always easy nor safe for the mother and the fetus. The guidelines recommend a target HbA1c of < 6.5% before and during pregnancy, a fasting blood glucose (BG) level of < 95 mg/dL, and 1-hour or 2-hour post-meal glucose levels of < 140 mg/dL and 120 mg/dL, respectively (9, 10). Self-monitoring of blood glucose (SMBG) using a finger stick glucose test or continuous glucose monitoring (CGM) device is critical for treatment adjustments. Nonetheless, tight glycemic control can increase the risk of hypoglycemia. Frequent hypoglycemia, particularly in T1DM, and hypoglycemia unawareness can further endanger both maternal and fetal health. Moreover, hypoglycemia can impair a patient’s adherence to treatment and complicate the condition further (9, 10).

Glycemic profiles (GP) have been studied in healthy women and women with GDM, showing that women with GDM had higher mean glucose levels and less time in the target range compared to those without GDM (11). The glycemic profile has also been compared between T1DM and T2DM during pregnancy, revealing that women with T2DM spend approximately 33% less time hyperglycemic and less time hypoglycemic (12). Studies comparing CGM devices with SMBG for monitoring glycemic control have shown similar outcomes, except in T1DM, where CGM appeared to be more effective (13, 14). The current guidelines recommend SMBG for monitoring pregnant women with HIP. At the same time, a CGM device is preferred for women with T1DM, who represent a minority of pregnant women with diabetes (10, 15). Since HbA1c is inaccurate for determining glycemic control during pregnancy for those with pre-existing diabetes and is not useful for the diagnosis and follow-up of GDM, SMBG is the most commonly used tool to guide treatment adjustments. To the best of the author’s knowledge, a real-world study on the glycemic profile, hypoglycemia events, and hypoglycemia awareness (HA) in pregnant women with various types of diabetes is lacking. 

## 2. Objectives

Therefore, this study aimed to observe and compare the GP, hypoglycemia events, and HA among women with GDM and pre-existing diabetes.

## 3. Methods

### 3.1. Study Design and Setting

The study participants were pregnant women with pre-existing diabetes and GDM referred to the specialized Diabetes Management Center (DMC) in Maternity Teaching Hospital, Sulaimaniyah, Iraq, from January to April 2024. Pre-existing diabetes had previously been diagnosed as T1DM, T2DM, or undetermined diabetes (either maturity-onset diabetes of the young or unknown type because they had no genetic testing or a clear diagnostic workup). The GDM patients were diagnosed using a 75 g glucose tolerance test (GTT) according to the NICE guidelines. Since a proportion of GDM patients were controlled with diet and lifestyle modifications, the registered GDM women in the DMC were those who needed medications or had uncontrolled glucose levels despite their treatment. The DMC team comprises a diabetologist, an obstetrician, a permanent doctor in obstetrics and gynecology, and a non-specialized nurse. The registered patients were briefly advised on controlling and monitoring their glucose levels, what action is required when the glucose levels become too low or high, and instructed to record them in their diaries. After registration, these patients had a 2- to 4-week appointment for glycemic control and obstetrical assessment throughout the pregnancy until post-puerperium. Of the 109 women with HIP, 103 patients agreed to participate in the study. Eventually, 100 patients with SMBG records were included in the final analysis, and three patients were excluded due to incomplete glycemic diaries.

#### 3.1.1. Inclusion Criteria

Pregnant women with gestational and preexisting diabetes who required antidiabetic medications and referral to a tertiary care center (DMC) were included.

#### 3.1.2. Exclusion Criteria

The exclusion criteria were patients with liver or renal impairment (except microalbuminuria), psychiatric disorders, and incomplete SMBG records (less than one week of diary entries).

### 3.2. Study Protocol

A standard-validated questionnaire was designed to collect data on the sociodemographic characteristics of the patients, their social history, diabetes history, and the history of other diseases that could affect the glycemic profile, such as psychiatric disorders and kidney and liver impairments. Information regarding pre-pregnancy planning, counseling, diabetes control and treatment, and hypoglycemia unawareness using the Gold Score Questionnaire (16), was also gathered. 

The registered pregnant women were instructed to monitor their blood glucose (BG) levels at least four times daily. For patients on metformin or once-daily intermediate/long-acting insulin, fasting glucose and postprandial glucose levels (1 hour after each meal) were advised. For women using multiple daily insulin injections (MDII), fasting, pre-meal, and 1-hour post-meal BG measurements were recommended.

Additionally, patients were strongly encouraged to record BG levels when experiencing symptoms of hypoglycemia and to take appropriate action to correct it. Treatment choices were reviewed and adjusted based on the patient’s general condition, capacity, obstetrical assessment, and SMBG records. The average daily BG records for each patient were calculated at each time point, including fasting and post-meal levels. A fasting/pre-meal glucose of 70 - 95 mg/dL was considered euglycemia, with any record above or below this range considered hyperglycemia or hypoglycemia, respectively. For 1-hour post-meal readings, any BG level between 70 and 140 mg/dL was considered euglycemia, while levels above that were labeled hyperglycemia, and any record below 70 mg/dL was classified as hypoglycemia. The proportions of in-range and out-of-range glycemia were reported. The GP reflected mean blood glucose levels at pre- and post-meals, and the proportion of eu-, hypo-, and hyperglycemic records.

The Gold score was used to assess HA based on an individual's response to the question: "Are you aware when your hypoglycemic episodes are commencing?" The score is derived from seven possible response options, with HA classified as follows: A score of ≤ 2 indicates normal awareness, meaning the individual can recognize the onset of hypoglycemia; a score of ≥ 4 indicates impaired awareness, suggesting difficulty recognizing hypoglycemic episodes; and a score of 3 is considered borderline, indicating partial awareness (16). Ultimately, the GP, hypoglycemia episodes, and HA were analyzed and compared between women with GDM and those with pre-existing diabetes.

### 3.3. Statistical Analysis

Statistical analysis was performed using SPSS version 27.0 (IBM SPSS Statistics). The normality of the data was assessed using the Kolmogorov-Smirnov and Shapiro-Wilk tests. Normally distributed continuous variables were expressed as the mean and standard deviation (SD). Differences in means between two groups were evaluated using an independent *t*-test, while one-way analysis of variance (ANOVA) was used for comparisons among three or more groups. For non-normally distributed continuous variables, data were summarized using the median and interquartile range (IQR). The Mann-Whitney U test was employed to assess differences between two groups, and the Kruskal-Wallis test was used for comparisons among three or more groups. Categorical data were presented as frequencies and relative frequencies. Chi-square and Fisher's exact tests were applied to examine differences between categorical variables across the study groups. The study had a power of 80%, meaning it was sufficiently powered to detect a significant difference with a sample size representing 44 participants in each group (GDM and pre-existing diabetes). A P-value of ≤ 0.05 was considered statistically significant.

## 4. Results

After reviewing the patients' data, three patients with incomplete glycemic data were excluded, and the data of 100 patients were analyzed. The majority of participants were aged over 35 years (49%), housewives (86%), residing in urban areas (74%), and had completed education at an institution or university (35%). Additionally, most were Kurdish (97%), non-smokers (99%), had no active lifestyle (57%), had 1 to 3 children (69%), and did not receive pre-pregnancy counseling (57%). More than half of the study participants had GDM (53%), while the remaining 47% had pre-existing diabetes: 5% T1DM, 36% T2DM, and 6% undetermined diabetes. Additionally, the majority had no medical conditions other than DM (77%), such as hypertension, thyroid disorders, tuberculosis, breast cancer, and ischemic heart disease. Moreover, the duration of pre-existing diabetes was <5 years in most cases, while a minority had DM for > 10 years. Regarding pregnancy planning and pre-pregnancy counseling, 47% of the pregnancies were planned, with 43% of those having received counseling, while the remaining 57% had not been counseled. None of the pre-existing diabetes patients received counseling from a team with triple specialties: A diabetologist, an obstetrician, and a nutritionist. However, 19.14% were counseled by both a diabetologist and an obstetrician. About a quarter (23.42%) were seen only by one member of the team, and 57.44% had not been counseled at all. Regarding women with GDM, about half had visited an obstetrician before their current pregnancy (Table 1). 

**Table 1.** Sociodemographic and Basal Characteristics of the Study Participants ^a^

Participant's Basal and Clinical Characteristics	Values
**Age (y)**	35.3 ± 5.9 (35.0)
21 - 27	14 (14)
28 - 35	37 (37)
> 35	49 (49)
**Occupation **	
Housewife	86 (86)
Employee	14 (14)
**Education**	
None	9.0 (9.0)
Primary	34 (34)
Secondary	22 (22)
Institution/university	35 (35)
**Residency**	
Urban	74 (74)
Suburban/rural	26 (26)
**Ethnicity**	
Kurd	97 (97)
Arab	3.0 (3.0)
**Smoking**	
Yes	1.0 (1.0)
No	99 (99)
**Exercise/day (min)**	
30	20 (20)
< 30	23 (23)
Never	57 (57)
**Medical conditions other than DM**	
Yes	33 (33)
No	77 (77)
**Parity**	
0.0	9.0 (9.0)
1 - 3	69 (69)
≥ 4	22 (22)
**Pre-pregnancy counselling**	
Yes	43 (43)
No	57 (57)
**Counselling team**	
Pre-existing DM	
Diabetologist	1.0 (1.0)
Obstetrician	10 (10)
Diabetologist and obstetrician	9.0 (9.0)
None	27 (27)
GDM	
Obstetrician	26 (26)
None	27 (27)
**Type of DM**	
Pre-existing DM	
T1DM	5.0 (5.0)
T2DM	36 (36)
Undetermined	6.0 (6.0)
GDM	53 (53)
**Duration of DM (y)**	
Pre-existing DM	
< 5	27 (27)
5 - 10	15 (15)
> 10	5.0 (5.0)
GDM	53 (53)
Total	100 (100)

Abbreviations: DM, diabetes mellitus; GDM, gestational diabetes mellitus; T1DM, type one diabetes mellitus; T2DM, Type 2 diabetes mellitus.

^a^ Values are expressed as mean ± SD (median) or No. (%).

The pre-pregnancy Body Mass Index (BMI) was almost similar between women with pre-existing DM (30.20 ± 5.35) and those with GDM (29.63 ± 3.77) (P = 0.54). Similarly, their body weight gain (kg) during the month of observation was also close (1.16 ± 1.1 in pre-existing DM and 1.13 ± 0.96, P = 0.67 in GDM). In contrast, pre-pregnancy HbA1c% and total daily insulin (TDI) dose injected during pregnancy were significantly higher in women with pre-existing DM compared to those with GDM (HbA1c%; 7.44 ± 1.42 vs 5.5 ± 0.43, P ≤ 0.001), (TDI units/kg; 0.52 ± 0.35 vs 0.24 ± 0.12, P ≤ 0.001). Most patients with GDM (71.7%) and pre-existing DM (97.9%) were treated with insulin with or without metformin (Table 2). 

**Table 2.** Metabolic Characteristics and Treatment in Women with Gestational Diabetes Mellitus and Pre-existing Diabetes ^a^

Participant’s Metabolic Characteristics and Treatment	Pre-existing DM	GDM	P-Value
**BMI (kg/m** ^**2**^ **)**	30.20 ± 5.35 (29.97)	29.63 ± 3.77 (29.30)	0.54 ^b^
**HbA1c% (pre-pregnancy)**	7.44 ± 1.42 (7.1)	5.5 ± 0.43 (5.6)	< 0.001 ^c^
**Weight change/current month (kg) **	1.16 ± 1.1 (1.2)	1.13 ± 0.96 (1.0)	0.67 ^c^
**Insulin (unit/kg/day)**	0.52 ± 0.35 (0.44)	0.24 ± 0.12 (0.22)	< 0.001 ^c^
**Metformin**	1.0 (2.1)	15 (28.3)	< 0.001 ^d^
**Insulin ± metformin**	46 (97.9)	38 (71.7)	-

Abbreviations, DM, diabetes mellitus; GDM, gestational diabetes mellitus; BMI, Body Mass Index.

^a^ Values are expressed as mean ± SD (median) or No. (%).

^b^ Independent *t*-test.

^c^ Mann–Whitney test.

^d^ Chi-square test.

**Figure 1.**
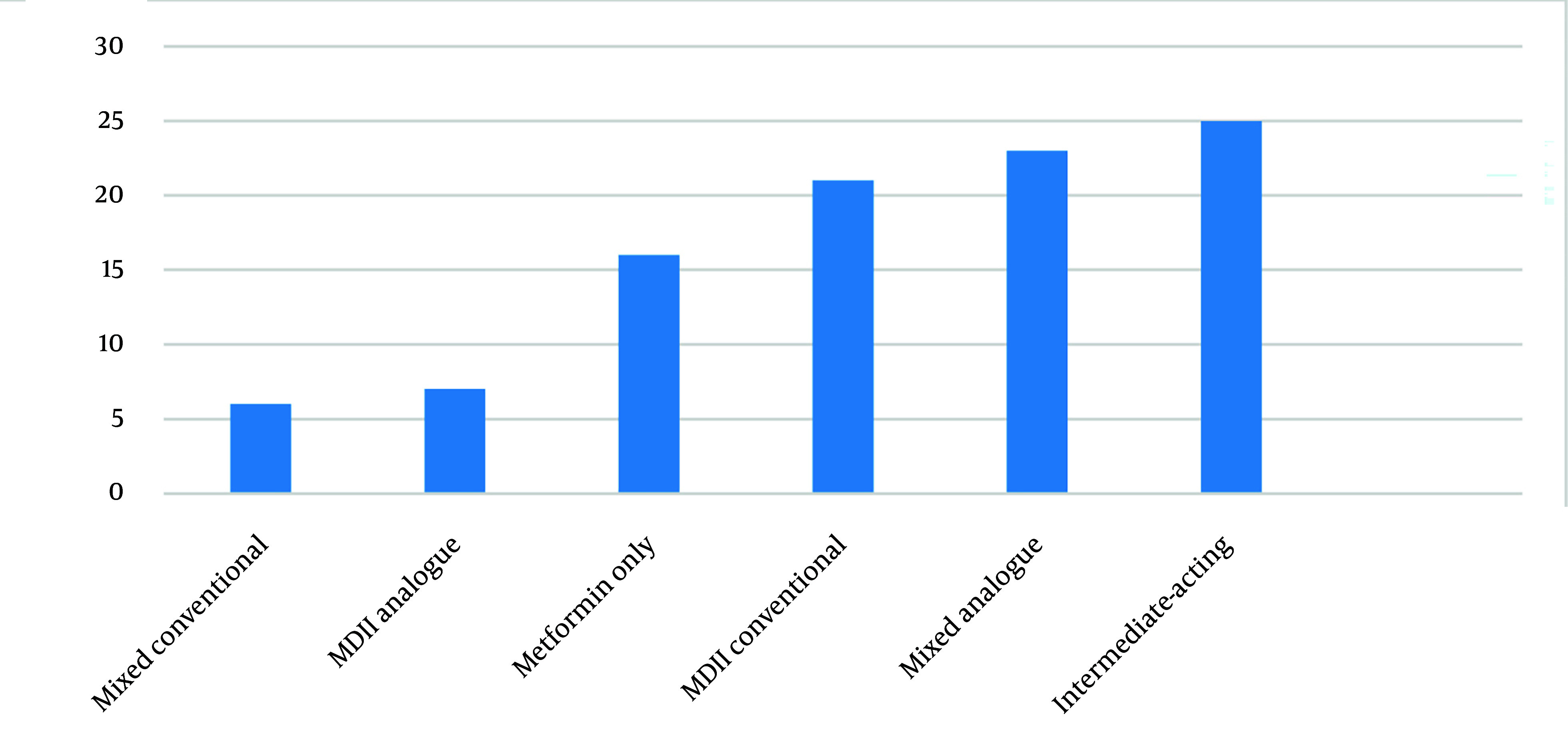
The proportion of pregnant women with hyperglycemia in pregnancy (HIP) on metformin and different insulin regimens, (Abbreviation: MDII, multiple daily injections of insulin).

Figure 1 shows the proportion of patients with their metformin and insulin regimen. The insulin types and regimens, ranked from most to least popular, were isophane insulin once or twice daily, followed by pre-mixed insulin analogue twice daily and conventional MDII. A minority of patients used MDII analogue or conventional pre-mixed insulin regimens.

The study participants recorded a total of 7055 BG levels, which means, on average, 68.49 records per patient. Fasting mean blood glucose (MBG) levels were significantly different between women with varying types of DM. The fasting MBG was highest in T1DM and lowest in GDM (P = 0.003), while at pre-lunch and pre-dinner, the MBG levels were numerically highest in T1DM and lowest in GDM (P = 0.08 and P = 0.60, respectively). At 1 hour after meals, the MBG levels were significantly different. It was highest in T1DM and lowest in GDM after breakfast and dinner (P = 0.002 and P = 0.009, respectively); however, the difference in MBG levels was not significant after lunch (P = 0.08) (Table 3). 

**Table 3.** Mean Blood Glucose Levels Before and One Hour After Meals in Different Types of Diabetes ^a^

Pre- and 1-Hour Post-meal MBG in Various Diabetes Types	T1DM (n = 5)	T2DM (n = 36)	Undetermined (n = 6)	GDM (n = 53)	P-Value
**Fasting BG**	111.89 ± 35.8	99.45 ± 17.4	112.28 ± 18.7	92.82 ± 10.6	0.003 ^b^
**Post-breakfast BG**	193.17 ± 76.3	140.78 ± 26.8	136.74 ± 17.1	132.01 ± 31.4	0.002 ^b^
**Before lunch BG**	131.32 ± 65.5	100.21 ± 18.5	125.15 ± 28.2	92.01 ± 11.2	0.08
**Post-lunch BG**	129.49 ± 47.5	141.78 ± 22.0	139.11 ± 20.44	129.70 ± 16.9	0.08
**Before dinner BG**	121.55 ± 51.4	108.22 ± 18.0	112.52 ± 27.4	103.27 ± 16.4	0.60
**Post-dinner BG**	144.81 ± 20.7	140.57 ± 19.3	139.49 ± 19.3	128.48 ± 16.3	0.009

Abbreviations: BG, blood glucose level; MBG, mean blood glucose; GDM, gestational diabetes mellitus; T1DM, type one diabetes mellitus; T2DM, type 2 diabetes mellitus.

^a^ Values are expressed as mean ± SD.

^b^ Significant difference using ANOVA test.

Table 4 shows that women with GDM had a significantly greater average proportion of euglycemic records than women with T2DM, undetermined diabetes and T1DM (64.4 ± 19.5, 49.5 ± 25.2, 40.9 ± 24.0 and 28.9 ± 24.0, respectively, p=0.001). In addition, women with GDM, had a significantly lower average proportion of hyperglycemic records than women with pre-existing diabetes (P = 0.01). The average proportion of hypoglycemic records was significantly greater in women with T1DM than in other types of diabetes (P = 0.001).

**Table 4.** Average Proportion of Hyperglycemia, Euglycemia and Hypoglycemia Records Among Patients

Pregnant Women with Diabetes	Number	Hyperglycemia	Euglycemia	Hypoglycemia
**T1DM**	5.0	54.4 ± 24.6 (64.2)	28.9 ± 24.0 (17.3)	16.7 ± 4.9 (17.6)
**T2DM**	36	48.3 ± 26.5 (44.4)	49.5 ± 25.2 (55.6)	2.2 ± 3.7 (0.0)
**Undetermined DM**	6.0	57.9 ± 24.5 (55.9)	40.9 ± 24.0 (43.6)	1.2 ± 1.6 (0.5)
**GDM**	53	34.0 ± 20.2 (30.3)	64.4 ± 19.5 (67.3)	1.6 ± 4.0 (0.0)
**P-value**	-	0.01	0.001	< 0.001

Abbreviations: GDM, gestational diabetes mellitus; T1DM, type one diabetes mellitus; T2DM, type 2 diabetes mellitus.

^a^ Values are expressed as mean ± SD (median) unless otherwise indicated.

^b^ They are performed using the Kruskal-Wallis test.

Table 5 shows that HE were significantly greater in women with pre-existing diabetes than in women with GDM (5.7 ± 9.7 vs 1.83 ± 4.9 events/patient/month, respectively, P = 0.002). According to diabetes types, HE was significantly greater among women with T1DM, followed by women with undetermined diabetes and T2DM, while it was lowest in women with GDM (29.2 vs 5.33 vs 2.5 vs 1.83 events/patient/month, respectively, P < 0.001).

**Table 5.** Hypoglycemia Events Per Patient Per Month in Pregnant Women with Hyperglycemia

Types of DM	Hypoglycemia Events/Patient/Month	P-Value
**T1DM**	29.2 ± 11.2 (30)	< 0.001
**T2DM**	2.5 ± 3.7 (0.5)	
**Undetermined DM**	5.33 ± 6.4 (3.0)	
**GDM**	1.83 ± 4.9 (0.0)	
**Pre-existing DM**	5.7 ± 9.7 (2.0)	0.002
**GDM**	1.83 ± 4.9 (0.0)	

Abbreviations: DM, diabetes mellitus; GDM, gestational diabetes mellitus; T1DM, type one diabetes mellitus; T2DM, type 2 diabetes mellitus.

^a^ Values are expressed as mean ± SD (median).

^b^ They were performed using the Mann–Whitney test.

On further analysis, all patients with T1DM had ≥ 4 episodes of hypoglycemia per month, whereas 33.3% of undetermined diabetes, 22.22% of T2DM, and 9.4% of GDM had ≥ 4 hypoglycemic episodes. The proportion of women without hypoglycemia was 0% in T1DM, 33.3% in undetermined diabetes, 50% in T2DM, and 67.92% in GDM, with the remaining proportions experiencing 1 - 4 hypoglycemic episodes per month (P < 0.001). The Gold score was analyzed to assess HA, and all women with GDM, T2DM, and undetermined DM were aware of hypoglycemia. Meanwhile, 20% of those with T1DM were fully aware of hypoglycemia, 40% were borderline, and 40% had impaired HA (P < 0.001) (Table 6). 

**Table 6.** Hypoglycemia and Hypoglycemia Awareness Among Women with Hyperglycemia ^a, b^

Variables	T1DM	T2DM	Undet-DM	GDM	P-Value
**Hypoglycemia episodes/month**					< 0.001
Never	0.0 (0.0)	18 (50)	2.0 (33.33)	36 (67.92)	
1 - 4	0.0 (0.0)	10 (27.77)	2.0 (33.33)	12 (22.64)	
> 4	5.0 (100)	8.0 (22.22)	2.0 (33.33)	5.0 (9.4)	
Total	5.0	36	6.0	53	
**Gold score (16)**					< 0.001
Normal	1.0 (20)	36 (100)	6.0 (100)	53 (100)	
Borderline	2.0 (40)	0.0 (0.0)	0.0 (0.0)	0.0 (0.0)	
Reduced HA	2.0 (40)	0.0 (0.0)	0.0 (0.0)	0.0 (0.0)	
Total	5.0	36	6.0	53	

Abbreviations: Undet-DM, undetermined diabetes; HA, hypoglycemia awareness.

^a^ Values are expressed as No. (%).

^b^ Performed by fisher exact test.

## 5. Discussion

This study reveals that approximately half of the referred pregnant women had preexisting diabetes, advanced maternal age, and unplanned pregnancies. Both the GDM and preexisting diabetes groups exhibited elevated pre-pregnancy BMI; however, insulin requirements were significantly greater among women with preexisting diabetes. Furthermore, the GDM group demonstrated a more stable GP, experiencing fewer episodes of hypoglycemia and maintaining intact HA. In contrast, women with preexisting T1DM exhibited the most unstable glycemic control and a reduction in HA.

Since the current study participants were referred women with diabetes, the proportion of GDM was smaller than what has been reported by observational studies (1, 2, 10, 17). Women with controlled GDM on lifestyle modification or metformin alone were managed at outpatient clinics and had yet to be referred to the tertiary center. Half of the participants were aged ≥ 35 years. Advanced maternal age is a risk factor for diabetes (18). Only a third of the participants were graduates, most of whom were housewives and city residents. Poor education and urbanization are associated with increased diabetes prevalence (19). The duration of pre-existing diabetes was < 5 years in the majority of cases, while a minority had diabetes for > 10 years. A long duration of diabetes can increase the risk of diabetes complications, which should be screened and managed to improve pregnancy outcomes (20, 21).

In the current study, more than half of the pregnancies were unplanned and uncounseled. Around half of the women with GDM had discussed their pregnancy plan with their obstetrician. Despite the importance of counseling, none of the women with pre-existing diabetes was counseled by a team with triple specialties: A diabetologist, an obstetrician, and a nutritionist, as recommended by the guidelines (9, 10). Notably, less than one-fifth of women with pre-existing diabetes were counseled by both a diabetologist and obstetrician; thus, the remaining women were counseled by an obstetrician or did not receive counseling at all. One-third of the pregnant women had other medical conditions, and a proportion of them had taken medications that were harmful during the critical weeks of embryogenesis, such as anti-tuberculosis drugs, angiotensin-converting enzyme inhibitors, and statins. Pregnancy planning and pre-pregnancy counseling are emphasized by the guidelines for all women, especially for women with pre-existing diabetes. Counseling aims to assess the risk factors, glucose control, associated diseases and complications, remove harmful medications, substitute them with appropriate alternatives, and monitor the pregnancy to reduce complications (9, 10, 22).

The pre-pregnancy BMI of women with pre-existing diabetes and GDM were similar, averaging around 30 kg/m². Increased body weight is a well-known risk factor for diabetes (18, 19). The mean weight gain during pregnancy was 1.1 kg/month, which is considered acceptable, as the recommended weight gain during the second and third trimesters for women with a similar pre-pregnancy BMI is 1 - 1.5 kg/month (23). As expected, pre-pregnancy HbA1c% and insulin requirements during pregnancy were significantly higher in women with pre-existing diabetes compared to those with GDM. In this study, a quarter of women with GDM were treated with metformin alone. In contrast, the majority of women with GDM and nearly all women with pre-existing diabetes were treated with insulin, with or without metformin.

Along with diet and lifestyle modification, insulin is the preferred treatment during pregnancy. However, some women may refuse insulin or may not be able to use it correctly, and in such cases, metformin is prescribed (9, 10). Isophane insulin, administered once or twice daily, was the most popular insulin regimen, followed by twice-daily pre-mixed insulin analogues and conventional MDII regimens. A minority used MDII-analogues or conventional pre-mixed insulin regimens. All women with T1DM were treated with either insulin analogues or conventional MDII. Although there is no consensus on the best insulin regimen during pregnancy, intermediate- or long-acting insulin for initiating treatment in GDM/T2DM patients and MDII for T1DM and uncontrolled GDM/T2DM patients seem to be logical options (24). Insulin analogues, such as Lispro, Aspart, Detemir, and Glargine, are preferred; however, more data are needed to confirm the safety of Glargine during pregnancy (9, 10, 24). The daily insulin requirement for the study participants, particularly women with GDM, was lower than recommended. This may be due to frequent follow-ups (every 2 - 4 weeks) and adherence to dietary and lifestyle advice, as around half of the women performed daily physical activities.

The GP were expressed as MBG levels before and after meals, as well as the proportions of euglycemic and non-euglycemic records. The MBG levels at fasting, post-breakfast, and post-dinner significantly differed between the diabetes types: Highest in T1DM, with a wider standard deviation, and lowest in GDM. At pre-lunch and pre-dinner, the MBG levels were numerically highest in T1DM and lowest in GDM. However, the MBG levels were similar after lunch. Nevertheless, frequent hypoglycemia in T1DM counterbalanced the hyperglycemic records, leading to a lower overall MBG level. This could explain why the difference was not significant at lunch and before dinner. Compared with GDM, patients with pre-existing diabetes, especially T1DM, showed greater glycemic instability (25, 26).

In line with other studies (12, 25), the proportion of euglycemic records was significantly higher in women with GDM than in women with pre-existing diabetes, with the lowest proportion of euglycemia observed in T1DM. Euglycemia is critical during pregnancy to reduce diabetes-associated risks. Nonetheless, maintaining near-normal glucose levels during pregnancy is challenging, particularly in women with pre-existing T1DM (12, 27). Regarding non-euglycemic records, about one-third of the BG records in GDM, versus more than half in pre-existing diabetes, were hyperglycemic. The proportion of hypoglycemia records was significantly greater in women with T1DM than in women with other types of diabetes. Hyper- and hypoglycemia should be appropriately managed, as every 5.0% increase in time-above-range or time-below-range glucose maintenance is associated with increased pregnancy complications (27). Thus, among the different types of diabetes, women with GDM had the greatest proportion of euglycemic records and the lowest proportion of out-of-range glucose records, while T1DM had the lowest proportion of euglycemic records and the greatest proportion of out-of-range glycemic records.

Another critical aspect of diabetes management is hypoglycemia. Hypoglycemic episodes were significantly more than three times higher in women with pre-existing diabetes than in women with GDM (5.7 vs. 1.83 events/patient/month). When HE was compared between different types of diabetes, the frequency of HE in T1DM was significantly more than five-fold, eleven-fold, and fifteen-fold greater than in T2DM, undetermined diabetes, and GDM, respectively. Compared to the present study, a previous study observed a slightly lower rate of mild HE in T1DM. However, the current research reported all HE with various degrees of severity (28). Based on the pathophysiology, HE is reported to be more common in T1DM than in T2DM (29). Studies documenting the frequency of HE of various severities in different types of diabetes during pregnancy are lacking, yet this information is crucial for predicting associated complications and taking appropriate action to prevent them. The number of HE is also important for predicting future HE. Among women with pre-existing diabetes, all women with T1DM, a third of those with undetermined diabetes, and nearly a quarter of those with T2DM had more than four HE per month, while this was reported by less than one-tenth of women with GDM. Analysis of the proportions of women without hypoglycemia records showed that none of the T1DM patients, a third of those with undetermined DM, half of those with T2DM, and two-thirds of those with GDM had no hypoglycemic records. Frequent hypoglycemia can adversely affect pregnancy and diabetes outcomes, reduce patient adherence to medications, and further deteriorate glycemic control (30-32).

Using the Gold score to observe the presence of HA among women with diabetes is an interesting approach. This study found that all women with GDM, T2DM, and undetermined DM were aware of hypoglycemia. In consistency with previous studies, about two-fifths of women with T1DM had reduced HA, while the remaining proportion had either normal or borderline HA (23, 30). 

Reduced HA should be taken seriously, as it significantly increases the risk of severe hypoglycemia, which could lead to adverse outcomes (2, 30-32). Reduced HA has been reported in a minority of non-pregnant insulin-treated patients with T2DM; however, its presence in pregnant women with T2DM is not well documented (32). The preservation of intact HA in non-T1DM pre-existing diabetes may be explained by the presence of functional insulin-counter-regulatory hormones and the short duration of diabetes, as the majority of these individuals had diabetes for less than five years. To the best of the author’s knowledge, no published studies have specifically examined HA in women with GDM and pregnant women with T2DM. These findings are novel and provide interesting insights into HA in women with GDM and T2DM. However, considering the short duration of diabetes, milder hyperglycemia, and the presence of insulin-counter-regulatory hormones in GDM, intact HA is expected in this population.

Since this is a real-world study, the findings represent almost actual GP of pregnant women with hyperglycemia who required referral to special care in our locality. To varying degrees, these results may be applicable to other populations, considering differences in healthcare practices, lifestyle factors, and demographics. Additionally, the limited sample size and the referral of patients to a tertiary center could influence the results. Although this study provides valuable insights into the GP and HA of pregnant women with different types of diabetes, further research is needed across diverse populations.

### 5.1. Strength and Limitations

The strengths of the present study can be summarized as follows. First, it provided insight into real-world practices and the challenges associated with HIP, highlighting how recommendations were applied, particularly in a country with a relatively high risk of diabetes and limited healthcare services. Second, for the first time, it compared GP, hypoglycemia episodes, and, importantly, HA among different types of diabetes during pregnancy. 

However, there are limitations to consider. Self-monitoring blood glucose cannot provide a comprehensive picture of glycemia throughout the day as CGM does, and the number of patients with T1DM and undetermined diabetes was small. Future research should take diabetes types into account when studying HIP.

### 5.2. Conclusions

This study found that approximately half of the referred pregnant women had pre-existing diabetes, advanced maternal age, and unplanned pregnancies. Both the GDM and pre-existing diabetes groups had elevated pre-pregnancy BMI; however, insulin requirements were significantly higher in women with pre-existing diabetes. The study observed significantly lower MBG levels in GDM and the highest MBG levels in T1DM compared to other types of pre-existing diabetes. Additionally, women with GDM had a significantly greater proportion of euglycemic records compared to those with pre-existing diabetes. Notably, hypoglycemia episodes per month were significantly lower in women with GDM than in those with pre-existing diabetes, with women with T1DM reporting the highest rate of hypoglycemia. These findings suggest that women with GDM had a more controlled GP than those with pre-existing diabetes. Among women with pre-existing diabetes, those with T1DM had the most unstable GP, experiencing frequent hypoglycemia and reduced HA. Maintaining normoglycemia during pregnancy is particularly challenging for women with pre-existing T1DM, who require individualized management plans.

## Data Availability

The dataset presented in the study is available on request from the corresponding author during submission or after its publication. The data are not publicly available due to ethical consideration.
